# Effect of miR-495 on lower extremity deep vein thrombosis through the TLR4 signaling pathway by regulation of IL1R1

**DOI:** 10.1042/BSR20180598

**Published:** 2018-12-21

**Authors:** Ke-Cheng Tang, Zhi-Peng Yang, Qiu Zeng, Jing Wang, Feng Guo, Yu Zhao

**Affiliations:** 1 Department of Vascular Surgery, The First Affiliated Hospital of Chongqing Medical University, Chongqing 400016, P.R. China; 2 Department of General Surgery, The Second Affiliated Hospital of Inner Mongolia Medical University, Hohhot 010030, P.R. China; 3 Department of Ultrasound, The First Affiliated Hospital of Chongqing Medical University, Chongqing 400016, P.R. China; 4 Department of Vascular Surgery, Universitätsmedizin Mannheim Theodor Kutzer Ufer, Mannheim 1-368167, Germany

**Keywords:** Cell cycle, Femoral vein, IL1R1, Lower extremity deep vein thrombosis, MicroRNA-495, TLR4 signaling pathway

## Abstract

Lower extremity deep vein thrombosis (LEDVT), a common peripheral vascular disease caused by a blood clot in a deep vein is usually accompanied by swelling of the lower limbs. MicroRNAs (miRs) have been reported to play roles in LEDVT. We aimed to investigate the effect of miR-495 on LEDVT via toll-like receptor 4 (TLR4) signaling pathway through interleukin 1 receptor type 1 (IL1R1). LEDVT mouse model was established, and the femoral vein (FV) tissues were collected to detect expressions of miR-495, IL1R1, and TLR4 signaling-related genes. The expressions of both CD31 and CD34 (markers for endothelial progenitor cells) in the FV endothelial cells as well as the proportion of CD31^+^/CD34^+^ cells in peripheral blood were measured in order to evaluate thrombosis. The effect of miR-495 on cell viability, cell cycle, and apoptosis was analyzed. IL1R1 was confirmed as the target gene of miR-495. Besides, inhibiting the miR-495 expression could increase IL1R1 expression along with activating the TLR4 signaling pathway. The total number of the leukocytes along with the ratio of weight to length of thrombus in the FV tissue showed an increase. The overexpression of miR-495 could promote FV endothelial cell viability. By injecting agomiR-495 and antagomiR-495 *in vivo*, the number of leukocytes in the FV tissues and the ratio of weight to length of thrombus were significantly decreased in the mice injected with the overexpressed miR-495, and the IL1R1/TLR4 signaling pathway was inhibited. Collectively, overexpressed miR-495 directly promotes proliferation while simultaneously inhibiting apoptosis of FV endothelial cells, alleviating FV thrombosis by inhibiting IL1R1 via suppression of TLR4 signaling pathway.

## Introduction

Thrombosis, usually present in the form of a blood clot, occurs in any part of the venous system, but frequently in the legs [1,2]. Lower extremity deep vein thrombosis (LEDVT) is both a common and serious peripheral vascular disease, triggering a chief morbidity along with mortality for limb loss, paradoxical embolization, pulmonary embolism, or post-thrombotic syndrome [3]. According to a recent study, LEDVT prevalence is approximately 1 out of 1000 adults every year [4]. Local pain, tenderness, edema, and swelling of the lower extremity are the most commonly found symptoms amongst LEDVT patients; however, over 30% of these cases present no signs or symptoms until an association is involved above the knee [5]. Although the Society of Interventional Radiology and the Society for Vascular Surgery has published quality improvement guidelines for the treatment of LEDVT and a relatively large amount of studies have reported a series of treatment options, no effective pharmacological methods that prevent thrombus-related damage to the vein wall in order to diminish the risk have been discovered [6]. It is common knowledge that inflammation is a major risk factor of LEDVT as levels of inflammatory cytokines are found to have increased in the peripheral blood of patients diagnosed with LEDVT [7]. Some microRNAs (miRs) have been proven to participate in inflammation activity along with having reports of acting as both prognostic and diagnostic indicators as well as the potential therapies in LEDVT [7–9].

The miR-495 has been identified as a tumor suppressor gene in various cancers, of which some demonstrated abnormal expression of miR-495 being reversely associated with multidrug resistance in non-small cell lung cancer as well as chronic myelogenous leukemia through targetting multidrug resistence (MDR1) or copper-transporting ATPase (ATP7A) [10]. Moreover, miR-495 has a mechanism that modulates the liver and pancreas development and regulates human umbilical vein endothelial cell proliferation and apoptosis through targetting CC chemokine ligand 2 (CCL2) [11,12]. It has been demonstrated that overexpression of miR-495 could lead to prohibition of LEDVT in peripheral blood [8]. Interleukin 1 receptor type 1 (IL1R1), located in a 470-kb cluster on 2q11, is predicted to be the target gene of miR-495 which is located on the bioinformatics website. The gene represents as a highly pertinent biological candidate, encoding protein that is known commonly as modulator of inflammatory processes linked with joint destruction, residing within a locus [13]. Other identifications were found that IL1R1 and toll-like receptor 4 (TLR4) share downstream opponents [14]. TLR4 plays a pivotal role in innate immunity through its property of activating an inflammatory signaling pathway [15]. Taken together, our investigation was designed with the purpose of verifying the effects of miR-495 targetting IL1R1 on LEDVT through the TLR4 signaling pathway.

## Materials and methods

### Ethics statement

The present study was carried out in strict accordance with the recommendations in the Guide for the Care and Use of Laboratory Animals of the National Institutes of Health. The protocol was approved by Ethics Committee of The First Affiliated Hospital of Chongqing Medical University.

### Model establishment

A total of 80 C57 specific pathogen-free (SPF) mice (weighing 30 ± 2 g) regardless of gender were purchased from the Animal Center of Kunming Medical University (Kunming, Yunnan, China). These mice were then housed under conditions ranging between 20 and 25°C with humidity up to 50–60% with free and unlimited access to both food and water. Two weeks later, mice were randomly assigned into a normal group (normal mice, *n*=20) and LEDVT group (model mice, *n*=60). Microvascular clamp of mice was used in order to block the bilateral femoral vein (FV) blood flow in order to prepare the mouse model. Surgical operations were all conducted on mice in the model group under sterile conditions. Then, a 3% pentobarbital sodium (mass ratio: 1 ml/kg, WS20051129, National Pharmaceutical Group Shanghai Chemical Reagent Co., Ltd., Shanghai, China) was injected into abdominal cavity of mice. Mice were then made to lay in supine position after shaving the bilateral inner thighs where their skin was cut longitudinally in order to expose the FV vessels at 2 cm, and then three parts of the vessels were clipped respectively with mosquito forceps to continue with the simulation. When the model was successfully established, the incision was sutured. These mice were fed normally after recovery. One day later, the swelling degree as well as the acral skin color was observed with no equipment, and those with obvious swelling in lower extremity and a violaceous-colored acral skin were appointed as experimental subjects.

A total of 50 mice were selected to participate in the *in vivo* experiments. After 1 week of adaptation, mice were then further assigned into the following groups: normal group (normal mice, *n*=10), blank group, LEDVT group (*n*=10), negative control (NC) group (LEDVT mice injected with agomiR-495 negative sequence, *n*=10), miR-495 inhibitor group (LEDVT mice injected with antagomiR-495 sequence, *n*=10), and miR-495 mimic group (LEDVT mice injected with agomiR-495 sequence, *n*=10). The injection concentration to the caudal vein was estimated to be 10 nmol [16].

### Hematoxylin–Eosin staining

On the 28th day, the chosen mice were both examined and killed. Thrombi of the mice were obtained after an incision into the medial thigh skin was made, followed by isolating FV vessels with the length and weight also calculated, and subsequently placed in a 10% neutral formaldehyde solution for 16–18 h. Later on, the fixed specimens were then dehydrated using gradient alcohol concentrations of 70, 80, 90, and 100%, cleared up with xylene, and immersed as well as embedded in paraffin. The embedded tissue blocks were further sectioned into 4-μm slices. These sections would later go on to be dewaxed with xylene, hydrated with the same gradient alcohol concentrations of 100, 90, 80, and 70%, with both distilled water and staining with Hematoxylin and 1% Eosin staining finishing the process. Next, the sections would be treated with alcohol depletion, cleared with xylene, and mounted with neutral resin. The ratios of weight to length in both the normal and model groups were compared. Under an optical microscope (LX51, Olympus, Japan), the number of leukocytes located in the thrombi was counted and the morphological differences of both cells and tissues between two groups were observed along with five positions selected in every field and analyzed by using an ImagePro Plus 6.0 software (IPP6.0, Media Cybernetics, Silver Springs, MD, U.S.A.).

### Reverse-transcription quantitative PCR

Total RNA was subsequently extracted from the mice FV tissues in each group with RNA extraction kit (Invitrogen Inc., Carlsbad, CA, U.S.A.). Primers of miR-495, IL1R1, NF-κB, interleukin 1 β (IL-1β), Rac family small GTPase 1 (Rac1), TLR4, U6, and glyceraldehyde-3-phosphate dehydrogenase (GAPDH) were both collectively designed and synthesized by the instructions provided by Takara Biotechnology Ltd. (Dalian, Liaoning, China) as presented in Table 1. The RNA was then reverse transcribed into cDNA using Prime Script RT kit with the system set as 10 μl. In accordance with manufacturer’s instructions, the reaction conditions were: reverse transcription for three times (15 min each time) at a temperature of 37°C and reverse transcriptase inactivation for 5 s at 85°C. The reaction liquid was conducted using a fluorescent quantitative PCR according to the SYBR® Premix Ex Taq™ II kit’s instructions. The reaction system was 50 μl composed of 25 μl of SYBR® Premix Ex Taq™ II (2×), 2 μl of PCR forward primer, 2 μl of PCR reverse primer, 1 μl of ROX Reference Dye (50×), 4 μl of DNA template, and 16 μl of ddH_2_O. Fluorescent quantitative PCR was carried out under the guidelines and technology of the ABI PRISM® 7300 system (ABI, Foster City, CA, U.S.A.) with reaction conditions indicated as the following: pre-denaturation at 90°C for 30 s, followed by 40 cycles of denaturation at 95°C for 5 s, and annealing/extension at 60°C for 30 s. U6 acted as an internal control for miR-495 and GAPDH for IL1R1, NF-κB, IL-1β, Rac1, and TLR4. Relative transcription levels for the target genes, including miR-495, IL1R1, NF-κB, IL-1β, Rac1, and TLR4, were all calculated using the 2^−ΔΔ*C*^_t_ method where Δ*C*_t_ = *C*_t__ target gene_ − *C*_tinternal control_, ΔΔ*C*_t_ = Δ*C*_texperimental group_ − Δ*C*_tcontrol group_. Experiment for each sample was repeated for a total of three times. This method was suitable for cell experiments.

**Table 1:** Primer sequence of miR-495, U6, IL1R1, TLR4, NF-κB p65, and IL-1β for reverse-transcription quantitative PCR

Genes	Sequences
*miR-495*	F: 5′-GGCAGGAAACAAACATGGTG-3′
	R: 5′-TCAACTGGTGTCGTGGAGTC-3′
*U6*	F: 5′-CTCGCTTCGGCAGCACA-3′
	R: 5′-AACGCTTCACGAATTTGCGT-3′
*IL1R1*	F: 5′-TGGAACAGAGCCAGTGTCAG-3′
	R: 5′-CAGGAGAAGTCGCAGGAAGT-3′
*TLR4*	F: 5′-CAGCAAAGTCCCTGATGACA-3′
	R: 5′-CCTGGGGAAAAACTCTGGAT-3′
*NF-κB p65*	F: 5′-GGTAGCCAAACAGGA-3′
	R: 5′-GAAGAGGGAAGAAGC-3′
*IL-1β*	F: 5′-AACCTGCTGGTGTGTGACGTTC-3′
	R: 5′-TTAGACAGCTGCACTACAGGCTCCGAGATG-3′
*Rac1*	F: 5′-GAGCAGAAGCTGATCTCCGAGGAG-3′
	R: 5′-TTACAACAGCAGGCATTTTCTCTT-3′
*GAPDH*	F: 5′-TTCACCACCATGGAGAAGGC-3′
	R: 5′-GGCATGGACTGTGGTCATGA-3′

Abbreviations: F, forward; NF-κB p65, nuclear factor-κ B p65; R, reverse.

### Western blot analysis

The mice’s FV tissues were then added along with liquid nitrogen and ground until reaching a uniformly fine powdered state followed by the addition of a protein lysate. The tissues were fully centrifuged (25764×***g***) at a temperature of 4°C allowing us to obtain the supernatant. The protein concentration was determined by the Bradford method. The extracted protein as well as the equal volume of 2× loading buffer was then mixed collectively and heated under 100°C for 5 min. The buffer was then used in association with 10% SDS/PAGE gel (20 µg in each lane). Electrophoresis was conducted and then the protein was transferred on to a PVDF membrane by the whole-wet method. The membrane would then be blocked in a 1× TBS with Tween 20 (TBST) containing 10% skimmed milk powder at room temperature and then further incubated overnight at 4°C along with rabbit anti primary antibodies including IL1R1 (ab106278, 1:500), TLR4 (ab13556, 1:500), nuclear factor-κ B p65 (NF-κB p65) (ab16502, 1:2000), pNF-κB p65 (ab86299, 1:2000), Rac1 (ab155938, 1:1000), IL-1β (ab106035, 1:2000), and GAPDH (ab9485, 1:2500). TBST washing was repeated for additional three times (5 min each time). Consequently, the membrane would then be incubated along with a secondary antibody of goat-anti-rabbit labeled by horseradish peroxidase (ab6721, 1:2000) for 2 h at room temperature, washed with TBST three times (5 min each time), and then developed with ECL agent (Beyotime Biotechnology Research Institute, Nanjing, Jiangsu, China). All antibodies mentioned above were provided by the Abcam Inc. (Cambridge, MA, U.S.A.). GAPDH served as the internal control. The gray value ratio of target bands to internal control bands would be regarded as the relative protein expression in cells. This method was suitable for cell experiments.

### Cell isolation, culture, and identification

FV vessels of LEDVT mice and normal mice were further washed with the PBS solution for a total of three times and placed in an axenic culture dish. The vessels were then cut longitudinally into small patches (2 mm × 2 mm), rinsed with D-Hank’s solution and additionally 1% of type I collagenase (Sigma, Santa Clara, CA, U.S.A.) for 10 min and were then centrifuged at 7×***g*** for 3 min. After the supernatant was removed, the aforementioned patches were added into the Roswell Park Memorial Institute (RPMI)-1640 (Gibco, Gaitherburg, MD, U.S.A.) culture medium containing 10% calf serum and incubated at 37°C with 5% CO_2._ When the cell adherence rate had reached 90%, a subculture cycle was performed. During the subculturing process, the nutrient fluid was cast while the medium was washed with PBS for a total of two times and treated along with 0.25% trypsin (Gibco, Gaitherburg, MD, U.S.A.). When the intercellular space had become enlarged, the trypsin was dumped with the cells being percussed into a single-cell suspension along with a cell culture medium; a routine culture and passage were both conducted. Under an inverted phase contrast microscope (GD-40, Olympus, Japan), the cellular morphology of the initial and the third generation was observed.

### Immunocytochemical staining

The expressions of related markers, such as CD31 and CD34, located in the vascular endothelial cells (VECs) of the third generation were determined by using immunohistochemistry. The VECs showing proper growth progression in the third generation were chosen and inoculated in order to be placed over a cover glass of a six-well plate. When cells grew to standard confluence, the cell culture medium was then injected overnight, washed with PBS solution, and placed along with 100% formaldehyde as well as smeared for 30 min. After washing the cells with PBS several times, primary antibodies of 100 μl rabbit polyclonal antibody CD31 (ab28364, 1:50, Abcam, Inc, MA, U.S.A.) and rabbit monoclonal antibody CD34 (ab81289, 1:2500, Abcam, Inc, MA, U.S.A.) were both added and water-bathed at 37°C for 1 h. After washing with PBS repeatedly, the cells were then added with a secondary antibody IgG (ab97051, 1:200, Abcam, Inc, MA, U.S.A.) labeled with biotin, water-bathed at 37°C for 30 min, and washed with PBS repeatedly. Diaminobenzidine (DAB) was then used in order to develop the color for 10 min. Cells then were washed with distilled water three times, stained with Hematoxylin–Eosin (HE) for 2 min and differentiated with 1% hydrochloric acid for 1 s. The dried and gluten-mounted cells were both observed and photographed under the microscope (PM-10A, Olympus, Japan).

### Dual luciferase reporter gene assay

The target gene of miR-495 was then predicted using the bioinformatics website (microRNA.org). IL1R1 was validated as being the direct target gene of miR-495 through use of a dual luciferase reporter gene assay. The overall length of the IL1R1 3′-UTR was artificially synthesized and the pmirGLO plasmid vector was transfected. Following transfection, IL1R1 3′-UTR mutant sequences as well as wild sequences lacking the miR-495 binding site were designed and inserted into the reporter vector. The pRL-TK vector expressing *Renilla* luciferase was meant to serve as the internal control. ECV304 cells in FV tissues of LEDVT had been co-transfected with both miR-495 mimic and recombinant plasmid of wild type (Wt-miR-495/IL1R1) or a mutant type (Mut-miR-495/IL1R1). The luciferase viability was then examined by the use of a dual luciferase reporter assay system (E1910, Promega Corp., Madison, Wisconsin, U.S.A.). After 48 h of transfection, the former culture medium had been absorbed, while the cells were then washed with PBS for a total of two times. Cells in each well were then added along with approximately 100 μl of passive lysis buffer and shaken gently at room temperature for 15 min with cell lysis buffer collected. The program was then set to pre-read for 2 s and read for 10 s. The volume of the LARIIStop&Glo® Reagent was also set at 100 μl each time. After the preparation of the LARII, both Stop & Glo®Reagent and light emitting tube or plate (20 μl from each sample) had been supplemented with cell lysis buffer and were placed in the bioluminescence detector. Fluorescence readings were obtained when the program was running and the data were reserved.

### Cell transfection and grouping

VECs of third generation in LEDVT mice and normal mice were obtained for further transfection and allocated into eight groups: normal (VECs obtained from normal mice), blank (VECs obtained from LEDVT mice without any sequence transfected), NC (VECs obtained from LEDVT mice transfected with miR-495 negative sequence), siRNA-NC (VECs obtained from LEDVT mice transfected with NC siRNA), miR-495 inhibitor (VECs obtained from LEDVT mice transfected with miR-495 inhibitor sequence), miR-495 mimic (VECs obtained from LEDVT mice transfected with miR-495 mimic sequence), IL1R1 siRNA (VECs obtained from LEDVT mice transfected with IL1RL siRNA) and miR-495 mimic + IL1R1 siRNA (VECs obtained from LEDVT mice co-transfected with miR-495 mimic and IL1R1 siRNA). These transfection sequences were purchased from Shanghai Gene Pharma Co., Ltd., (Shanghai, China). The transfected cells were then inoculated into a 50-ml culture flask and endured growth between 30 and 50% confluence in a complete medium. Lipofectamine 2000 (Invitrogen, CA, U.S.A.) as well as the DNA (5 µl Lipofectamine 2000 + 100 µl serum-free nutrient solution) was prepared in sterile eppendorf (EP) tubes and placed at room temperature for 5 min. The siRNA (50 nmol) + serum-free nutrient solution (100 µl) would then be placed for an additional 20 min under room temperature conditions in order to make the combination of liposome and DNA into a complex. The cells in the culture flask were washed using a serum-free nutrient solution. The complex was added along with a nutrient solution without either serum or antibiotic, mixed under mild conditions, and finally added in 50 ml culture flask for further transfection. Subsequently, the mixture was cultured at 37°C with 5% CO_2_ for 6–8 h. After the complete medium was added, the culturing continued for an additional 24–48 h and the cells were subsequently collected.

### MTT assay

The transfected cells were washed under PBS solution twice when the growth density was approximately 80%. The cells were then treated with 0.25% trypsin in order to make single-cell suspension. After the number was counted, the newly constructed cell suspension was seeded into a 96-well plate with a set of 3 × 10^3^ to 6 ×10^3^ cells in each well. Six replicate wells were prepared with the volume of each well set at 200 μl. After culture, 20 μl of MTT solution (5 mg/ml, Sigma–Aldrich Chemical Company, St. Louis, MO, U.S.A.) was added into each well, with the plate incubating for 4 h. With the culture medium having been aspirated, 150 μl of DMSO (Sigma–Aldrich Chemical Company, St. Louis, MO, U.S.A.) was further added into each well. After gently shaking for 10 min, the optical density (OD) at 450 nm was measured using an ELISA reader (Nanjing Iron & Steel Co., Ltd., Nanjing, Jiangsu, China). The cell viability curve was pictured with time acting as the horizontal ordinate and OD value as the ordinate [17].

### Flow cytometry

Forty-eight hours following cell transfection, the cells were collected, treated with 0.25% trypsin solution, and adjusted to 1 × 10^6^ cells/ml. Then, 1 ml cells were centrifuged at 402×***g*** for 10 min, followed by abandoning the supernatant. The cells, following centrifugation, were collected with every 1 ml added along with 2 ml of PBS, and then further centrifuged. After the supernatant was dropped, the cells were placed in a pre-cooled ethanol solution overnight at 4°C. On the next day, the fixed cells were then washed with PBS for additional two washes. Then, 100 μl of cell suspension with no less than 10^6^ cells/ml was taken, added with 1 ml of propidium iodide (PI, 50 mg/l) staining liquor (containing RNAase), and placed in an environment avoiding any exposure to light for 30 min. Following the 30 min of non-light exposure, the cell suspension was filtered along with 100-mesh nylon mesh. The flow cytometry (Beijing Origin Technology Co., Ltd., Beijing, China) was utilized in order to record any evident red fluorescence light of excitation wavelength at 488 nm further determining the cell cycle.

Cell apoptosis was examined via Annexin V-FITC/PI double staining method. The cells were then treated in the manner similar to cell cycle examination. Cells were then collected following 48 h of incubation at a temperature of 37°C with 5% CO_2_, washed under a PBS solution for two times, centrifuged, and re-suspended in 200 μl binding buffer. Next, the cells were further mixed with 10 μl Annexin V-FITC and 5 μl PI gently, reacted in a manner that avoided exposure to light at room temperature, and then added along with 300 μl binding buffer. The cell apoptosis was then measured according to the activation of wavelength at 488 nm through flow cytometry.

Both CD31 and CD34 (Becton Dickinson Biosciences, San Jose, CA, U.S.A.) double-labeled staining was used in order to detect endothelial cells. Ten mice in both the normal and the DVT groups were selected in order to take 500 μl of whole blood via a heart puncture. The whole blood of 200 μl was then added to the heparin anticoagulant tube, added with 20 μl CD31 and CD34 antibodies, and incubated for 30 min on the ice avoiding any exposure to light. Then, the tube was added with 2–3 ml red cell lysate, incubated for 3 min at room temperature, washed once with PBS, and suspended with PBS after centrifugation with the supernatant removed. Finally, the flow cytometry was conducted [18,19].

### Statistical analysis

Statistical analysis was conducted by SPSS 21.0 software (IBM Corp., Armonk, NY, U.S.A.). Results were expressed using the mean ± S.D. Differences between two groups were compared by *t* test, and comparison amongst multiple groups was compared by one-way ANOVA. *P*<0.05 was considered statistically significant.

## Results

### Higher ratio of thrombus weight to length and more leukocytes in LEDVT mice

HE staining method was used in order to observe the FV morphology of FV tissues in both the normal group and the model group. In normal FV tissues, structure and morphology were presented as complete, endothelial cells were in fusiform shape with both a regular arrangement and even size; vascular smooth muscle of middle membrane was also arrayed in order and all surface of endangium was smooth. After model establishment, partial VECs were exfoliated with the structure of vascular smooth muscle in disorder, while plenty of inflammatory cells on vascular wall and tissue space were involved with focal infiltration; the vascular wall was further thinning and the thrombi had been spread to most parts of lumen. In comparison with the normal group, the ratio of weight to length of thrombi as well as the number of leukocytes was increased in mice of the model group (*P*<0.05) (Supplementary Figure S1A–C).

### miR-495 expression is decreased, IL1R1 expression is increased, and the TLR4 signaling pathway is activated after LEDVT

**Figure 1:**
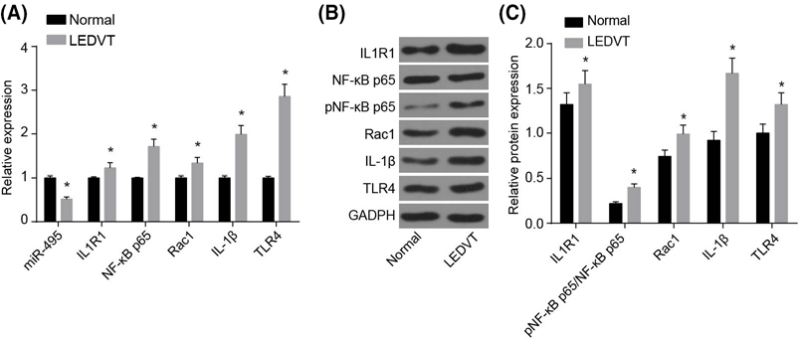
miR-495 expression is decreased, IL1R1 expression is increased, and the TLR4 signaling pathway is activated after LEDVT (**A**) Relative miR-495 expression is decreased and mRNA expressions of IL1R1, NF-κB, p65, Rac1, IL-1β, and TLR4 are increased after DVT; (**B**,**C**) relative protein expressions of IL1R1, NF-κB p65/pNF-κB p65, Rac1, IL-1β, and TLR4 are increased after DVT; *, *P*<0.05 compared with the normal group. Abbreviation: pNF-κB p65, p-nuclear factor-κ B p65.

Then, reverse-transcription quantitative PCR (RT-qPCR) and Western blot analysis were used in order to detect the miR-495 expression and mRNA and protein levels of IL1R1, and the TLR4 signaling related genes in FV tissues in normal as well as LEDVT mice. When comparing with the normal group, the relative expression of miR-495 in FV tissues of LEDVT mice had been reduced (*P*<0.05), whereas the relative mRNA expressions of IL1R1, NF-κBp65, Rac1, IL-1β, and TLR4 were all enhanced (all *P*<0.05, Figure 1A). In comparison with the normal group, FV tissues of the LEDVT mice showed much higher protein expressions in all of NF-κB p65, Rac1, IL-1β, and TLR4 (all *P*<0.05, Figure 1B,C). These results demonstrated that down-regulation of miR-495 in FV tissues of LEDVT mice led to the activation of the TLR4 signaling pathway.

### Higher positive expression of CD31 and CD34 after LEDVT in VECs

Primary VECs presented with fusiform or polygonal rows, mononuclear with a relatively large nucleus visible under the microscope (100×). Cells of the third generation were still in polygon shape with a relatively large nucleus in the center featuring an obvious ‘slab stone’ shape while a mosaic connection was observed amongst cells. It had been confirmed that the cultured cells after isolation were VECs (Supplementary Figure S2A,B).

**Figure 2:**
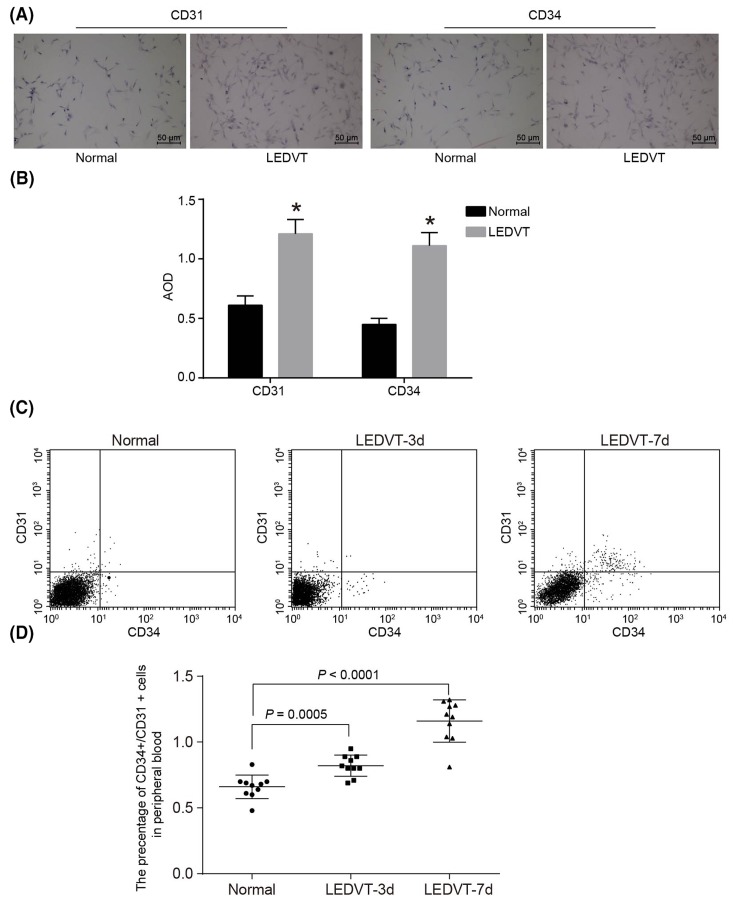
Higher positive expression of CD31 and CD34 after LEDVT in VECs Cells with positively expressed CD31 and CD34 are stained pale brown in the LEDVT group where CD31 and CD34 show positive expressions. As for VECs in the normal group, some areas are stained lightly or even not stained. (**A**) Immunocytochemical staining of CD31 and CD34 in endothelial cells *in vitro*; (**B**) AOD of CD31 and CD34 in endothelial cells; (**C**) proportion of CD31 and CD34 positive cells in peripheral blood; (**D**) statistical analysis of proportion of CD31 and CD34 positive cells in peripheral blood. Abbreviation: AOD, average optional density.

Both CD31 and CD34 positive cells can be considered as markers for endothelial progenitor cells. In both LEDVT patients and LEDVT modeled mice, the CD31^+^CD34^+^ cells in peripheral blood had increased dramatically and recruited to the venous thrombosis, which will help in recanalization of the thrombus. Therefore, recognizing the previous information, we detected the expressions of CD31 and CD34 in the cultured endothelial cells *in vitro*, and detected the number of CD31^+^CD34^+^ in the peripheral blood of LEDVT modeled mice by flow cytometry. Immunocytochemical staining was used in order to detect the expressions of both CD31 and CD34 in VECs. Cells with positively expressed CD31 and CD34 were stained pale brown in the LEDVT group with both CD31 and CD34 showing positive expressions. As for the VECs in the normal group, some areas were stained lightly or even not stained altogether. In comparison with the normal group, both CD31 and CD34 in the LEDVT group were expressed positively with a significantly higher positive rate reflecting stronger positive degree (*P*<0.05) (Figure 2). In comparison with the normal group, the number of CD31 and CD34 positive cells in the peripheral blood of mice increased significantly on the 3rd and 7th days following LEDVT modeling construction (*P*<0.05).

### IL1R1 is the target gene of miR-495

**Figure 3:**
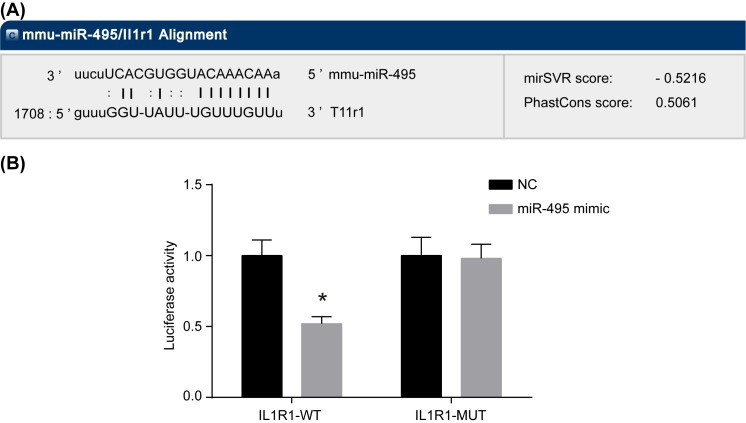
IL1R1 is the target gene of miR-495 (**A**) Predicted miR-495-binding sites on the IL1R1 3′-UTR; (**B**) luciferase activity of IL1R1-WT transfected with miR-495 mimic is significantly decreased, indicating that IL1R1 is the target gene of miR-495; *, *P*<0.05 compared with the NC group.

Predicting miR-495-binding sites on the IL1R1 3′-UTR was confirmed by using the previously mentioned bioinformatics website (microRNA.org) (Figure 3A). A dual luciferase reporter gene assay, miR-495 mimic had shown no obvious influence on the luciferase activity of Mut-miR-495/IL1R but resulted in a reduction in 8367 luciferase activity of Wt-miR-495/IL1R1 along with possible statistical significance (*P*<0.05, Figure 3B). Based on all the aforementioned information, miR-495 was found to have been able to inhibit IL1R1.

### Overexpression of miR-495 inhibits the TLR4 signaling pathway via down-regulating IL1R1 expression

**Figure 4:**
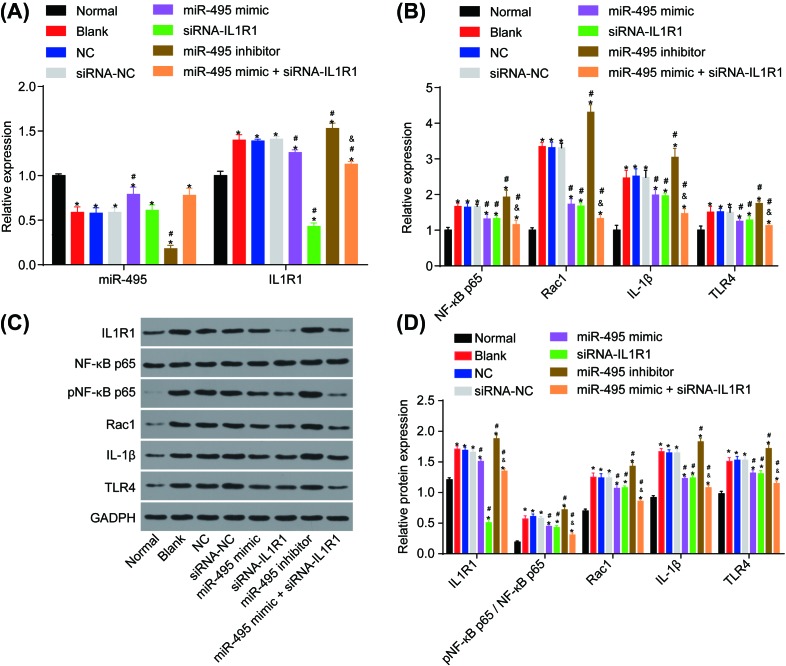
Overexpression of miR-495 inhibits the TLR4 signaling pathway via down-regulating IL1R1 expression (**A**) mRNA expressions of IL1R1 and miR-495; (**B**) mRNA expressions of the TLR4 signaling pathway related genes; (**C**) protein expressions of IL1R1 and the TLR4 signaling pathway related genes; (**D**) statistical analysis of protein expressions of IL1R1 and the TLR4 signaling pathway related genes; *, *P*<0.05 compared with the normal group; ^#^, *P*<0.05 compared with the blank, NC, and siRNA-NC groups; ^&^, *P*<0.05 compared with the miR-495 mimic and siRNA-IL1R1 groups.

Because both IL1R1 and TLR4 share some downstream signaling pathways, we wanted to determine whether or not miR-495 regulates the TLR4 signaling pathway through IL1R1. RT-qPCR and Western blot analysis were used in order to detect the expressions of miR-495, IL1R1, and the TLR4 signaling related genes in VECs. In comparison with the normal group, the miR-495 expression was reduced, while both mRNA and protein expressions of IL1R1, TLR4, NF-κB p65, Rac1, and IL-1β were enhanced in each group following cell transfection (all *P*<0.05). In the blank, NC, and siRNA-NC groups, expressions of miR-495, IL1R1, TLR4, NF-κB p65, Rac1, and IL-1β demonstrated no obvious difference (*P*>0.05). In comparison with the blank, NC, and siRNA-NC groups, the mRNA and protein expressions of IL1R1, TLR4, NF-κB p65, Rac1, and IL-1β had shown elevation in the miR-495 inhibitor group (all *P*<0.05), whereas the miR-495 expression had declined (*P*<0.05). Both mRNA and protein expressions of IL1R1, TLR4, NF-κB p65, Rac1, and IL-1β decreased in groups transfected with miR-495 mimic, siRNA-IL1R1, and miR-495 mimic + siRNA-IL1R1 (all *P*<0.05), while the miR-495 expression in the miR-495 mimic and miR-495 mimic + siRNA-IL1R1 groups both increased (all *P*<0.05). MiR-495 expression in the siRNA-IL1R1 group had also shown no significant difference (*P*>0.05). The miR-495 mimic + siRNA-IL1R1 group showed a decreased combination of mRNA and protein expressions of IL1R1, NF-κB p65, Rac1, IL-1β and TLR4 when comparing with the miR-495 mimic and siRNA-IL1R1 groups (*P*<0.05, Figure 4A–D). Overall, overexpression of miR-495 inhibited the TLR4 signaling pathway via down-regulating IL1R1 expression.

### Up-regulated miR-495 and down-regulated IL1R1 promote FV cell viability

**Figure 5:**
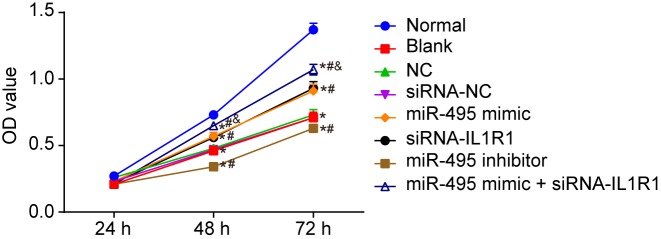
Overexpression of miR-494 and silenced IL1R1 increase cell viability detected by MTT assay. *P<0.05 compared with the normal group; ^#^*P*<0.05 compared with the blank, NC, and siRNA-NC groups; ^&^*P*<0.05 compared with the miR-495 mimic and siRNA-IL1R1 groups.

MTT assay was utilized in order to detect the viability of the FV cells. No significant difference involving the cell proliferation ability was found in each group at the 24-h mark (*P*>0.05), but at both 48 and 72 h, other transfected groups started showing an inhibited cell proliferation ability when compared with the normal group (all *P*<0.05). The ability of cell proliferation had also shown no significant differences in the blank, NC, and siRNA-NC groups (*P*>0.05). In comparison with the blank, NC, and si-RNA-NC groups, the proliferation ability of FV cells had become strengthened (all *P*<0.05) in the miR-495 mimic, siRNA-IL1R1, and miR-495 mimic + siRNA-IL1R1 groups, but suppressed in the miR-495 inhibitor group; while no significant differences were found between the miR-495 mimic and siRNA-IL1R1 groups (*P*>0.05). In comparison with both the miR-495 mimic and siRNA-IL1R1 groups, proliferation ability of VECs was also found to have been enhanced in the miR-495 mimic + siRNA-IL1R1 groups (*P*<0.05) (Figure 5). These findings lead us to believe that up-regulating miR-495 and down-regulating IL1R1 promoted FV endothelial cell viability.

### Up-regulated miR-495 and silenced IL1R1 promote cell cycle entry in FV cells

**Figure 6:**
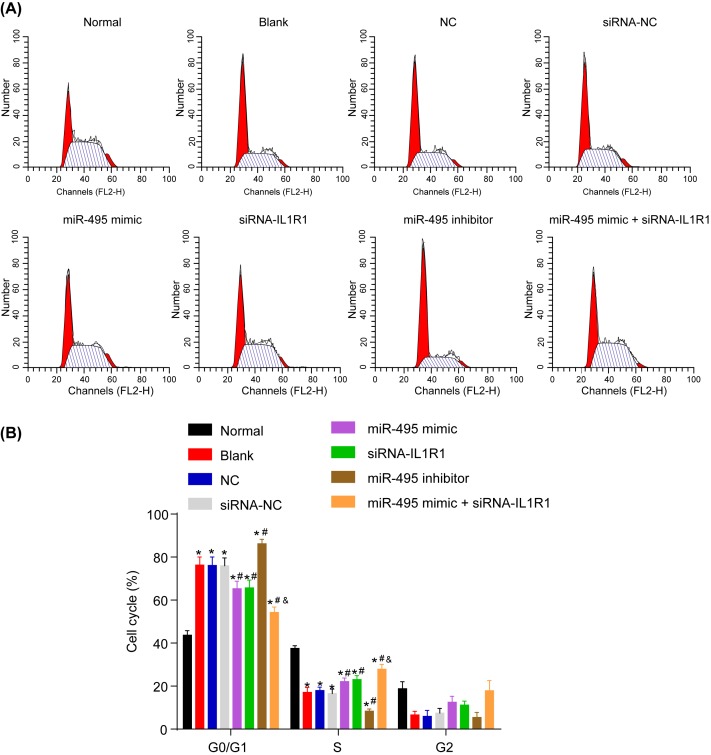
Cell cycle in each group after transfection detected by flow cytometry (**A**,**B**) Cells transfected with miR-495 mimic, siRNA-IL1R1 and miR-495 mimic + siRNA-IL1R1 mostly stayed in S phase; *, *P*<0.05 compared with the normal group; ^#^, *P*<0.05 compared with the blank, NC, and siRNA-NC groups; ^&^, *P*<0.05 compared with the miR-495 mimic and siRNA-IL1R1 groups.

Flow cytometry was used in order to detect the cell cycle. G_0_/G_1_ phase lengthened and the S phase shortened in each group after transfection when comparing with the normal group (all *P*<0.05). No significant differences were observed in the blank, NC, and siRNA-NC groups (all *P*>0.05). When comparing with the three previously mentioned groups, the G_0_/G_1_ phase lengthened and S phase shortened in the miR-495 inhibitor group (all *P*<0.05); G_0_/G_1_ phase shortened and S phase lengthened in the groups transfected with miR-495 mimic, siRNA-IL1R1, and miR-495 mimic + siRNA-IL1R1; no significant differences were found between the miR-495 mimic and siRNA-IL1R1 groups (*P*>0.05). In comparison with the two groups mentioned above, the G_0_/G_1_ phase was shorter and S phase was longer in the miR-495 mimic + siRNA-IL1R1 group (*P*<0.05) (Figure 6A,B).

### Up-regulated miR-495 and silenced IL1R1 inhibit apoptosis in FV cells

**Figure 7:**
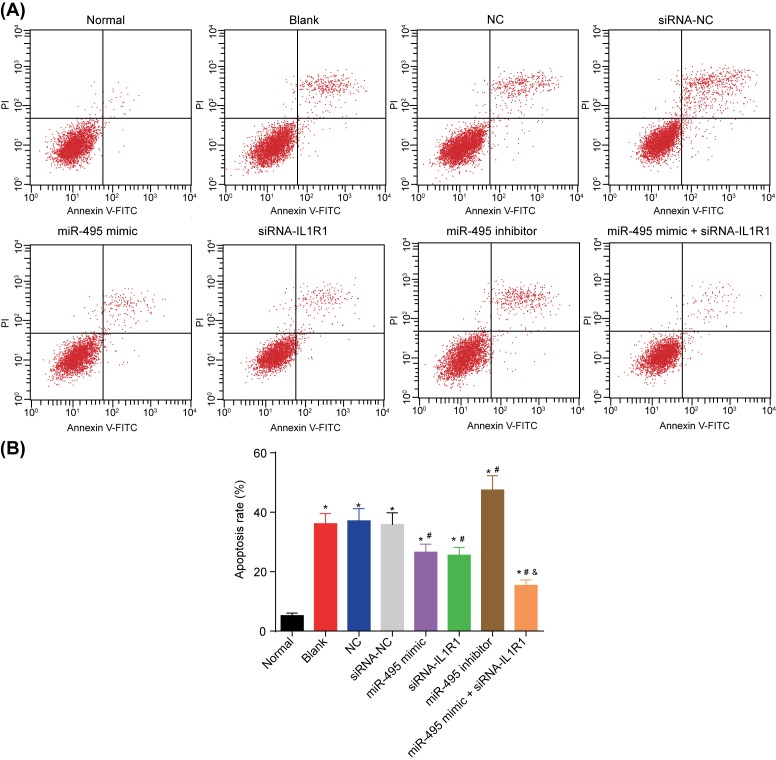
Up-regulated miR-495 and silenced IL1R1 inhibit apoptosis in FV cells (**A**) Flow pattern of cell apoptosis; (**B**) histogram of cell apoptosis; *, *P*<0.05 compared with the normal group; ^#^, *P*<0.05 compared with the blank, NC, and siRNA-NC groups; ^&^, *P*<0.05 compared with the miR-495 mimic and siRNA-IL1R1 groups.

In order to measure the cell apoptosis, we adopted the use of the PI staining method. Early metaphase apoptotic cells had been illustrated in the right lower quadrant, with both late apoptotic cells and secondary necrosis cells presented in the right upper quadrant, mechanically damaged or necrosis cells as well as living cells also present in the left lower quadrant. In comparison with the normal group, the cell apoptosis following transfection increased (all *P*<0.05). No significant difference involving the cell proliferation rate was found in the blank, NC, and siRNA-NC groups (*P*>0.05); while comparing with these groups, the cell apoptosis rate enhanced in the miR-495 inhibitor group and fell in the miR-495 mimic, siRNA-IL1R1, and miR-495 mimic + siRNA-IL1R1 groups (all *P*<0.05). Moreover, no significant differences were detected between both the miR-495 mimic and siRNA-IL1R1 groups (*P*>0.05). In comparison with both the miR-495 mimic and siRNA-IL1R1 groups, the miR-495 mimic + siRNA-IL1R1 group had shown a decreased cell apoptosis rate (*P*<0.05) (Figure 7A,B). Altogether, an overexpression of miR-495 and down-regulation of IL1R1 proved to inhibit the FV endothelial cell apoptosis.

### MiR-495 is involved in regulating IL1R1/TLR4 signaling pathway in LEDVT model

**Figure 8:**
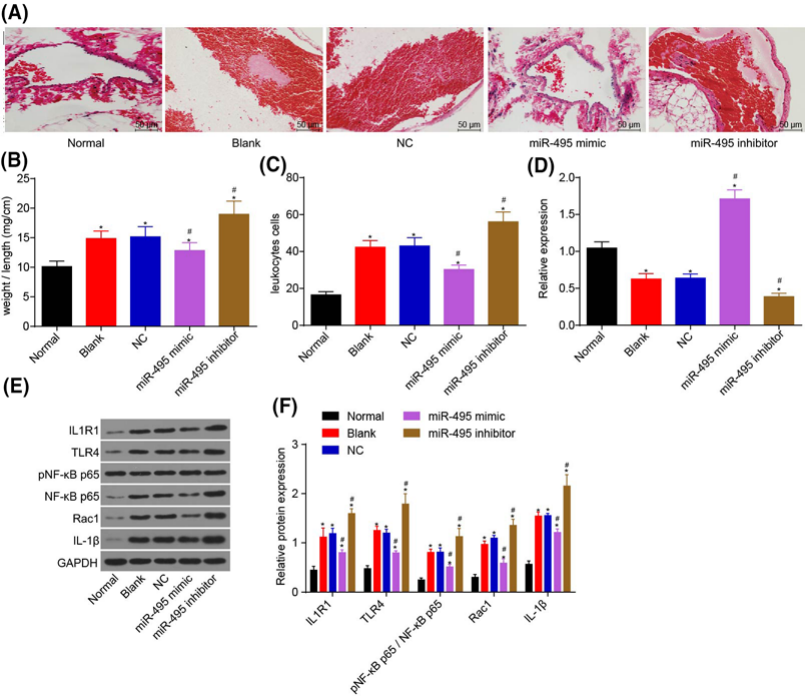
MiR-495 inhibits the number of inflammatory leukocytes in the FV tissues and the ratio of weight to length of thrombus by inhibiting IL1R1/TLR4 signaling pathway (**A**) HE staining was used to detect the pathological changes of FV thrombosis in mice; (**B**) a statistical map of the ratio of weight to length of thrombus in each group; (**C**) the statistical map of the number of leukocytes in each group of thrombus; (**D**) miR-495 expression in the plasma of each group was detected by RT-qPCR; (**E**,**F**) protein expressions of IL1R1 and TLR4 signaling-related genes in venous thrombosis tissues by Western blot analysis; *, *P*<0.05 compared with the normal group; ^#^, *P*<0.05 compared with the blank, NC, and siRNA-NC groups.

After successful construction of the LEDVT model and the injection of both agomiR-495 and antagomiR-495 into the tail vein of mice, HE staining was used in order to observe the histopathological changes in the FV in the two groups as well as to measure the ratio of weight to length of thrombus along with the number of leukocytes in the thrombus. The RT-qPCR method was used so as to detect the expression of miR-495 in the plasma of each group, and expressions of IL1R1, NF-κB p65, pNF-κB p65, Rac1, IL-1β, and TLR4 in tissues were detected via Western blot analysis. In comparison with the blank group and the NC group, the ratio of weight to length of thrombus decreased significantly, and the number of inflammatory leukocytes in the FV also significantly decreased in the miR-495 mimic group (all *P*<0.05); while the thrombus of the miR-495 inhibitor mice spread all the way to the majority of the lumen, and the ratio of weight to length of thrombus along with the number of inflammatory leukocytes in FV tissue increased significantly (*P*<0.05) (Figure 8A–C). When comparing with the blank and NC groups, the level of miR-495 located in the plasma increased in the miR-495 mimic group (*P*<0.05); however, the level of miR-495 in the plasma of the miR-495 inhibitor mice decreased (*P*<0.05). The results of Western blot analysis provided to us that, when compared with the blank and NC groups, the levels of IL1R1, pNF-κB p65, Rac1, IL-1β, and TLR4 in the miR-495 mimic group were significantly lower; while the levels of IL1R1, pNF-κB p65, Rac1, IL-1β, and TLR4 in the miR-495 inhibitor group had significantly increased (*P*<0.05) (Figure 8 D–F). These results suggested that the number of leukocytes in the FV tissues and the ratio of weight to length of thrombus were significantly decreased by overexpressing miR-495 to inhibit the activation of the TLR4 signaling pathway.

## Discussion

As revealed throughout our experiment, the overexpression of miR-495 promotes proliferation as well as inhibits the apoptosis of FV endothelial cells, IL1R1 leads to inactivation of TLR4 signaling pathway through negative regulation and promotes thrombolysis of LEDVT. Decreased expression of miR-495 was shown in the plasma as well as the FV tissues of LEDVT mice, in which expressions of IL1R1 and the TLR4 signaling pathway related genes increased.

A previous investigation conducted by Jin et al. [20] also mentioned that the regulatory association between miR and mRNA suggested they had played key roles through endothelial cells in thrombosis. In fact, the development of thrombosis has a significant relevance with cancers; for example, cancer is a commonly associated risk factor for LEDVT patients [21]. Increased levels of leukocytes, platelets, and tissue factor-positive macrovesicles are all potential elements either alone or in combination with advance cancer-associated thrombosis [22]. Interestingly, miR-495 has been found in a variety of cancers including breast cancer, gastric cancer, and non-small cell lung cancer cells [23–25]. Additionally, in line with our study, Li et al. [26] also found decreased miR-495 expression in peripheral blood of patients with DVT, and miR-495 inhibited LEDVT in peripheral blood via suppression of Stat3. It has also been suggested that IL1R1 is involved in the pathogenesis of hand, hip, and knee osteoarthritis [27]. What was more, up-regulation of TLR4 may be a potential biomarker of inflammatory joint pain through myeloid differentiation primary response (MyD88) signaling pathway [28]. TLRs are also vital factors in the innate immune system and induce an inflammatory response to foreign pathogens, including viruses, bacteria, and fungi [29]. A very recent study also discovered a similar result of LEDVT rats showing higher expressions of NF-κB, Rac1, IL-1β, and TLR4 after lower miR-335-5p expression [9].

As illustrated throughout our study, the role of miR-495 in LEDVT was carried out through the TLR4 signaling pathway. We discovered that an overexpression of miR-495 inhibits the TLR4 signaling pathway via down-regulating IL1R1 expression. As the dual-luciferase reporter gene assay verified, *IL1R1* is a target gene of miR-495. The *IL1R1* gene encodes receptor whose activation by the binding of a specific ligand leads to the activation of NF-κB, which is known as a modulator of inflammatory and immune gene expression [13]. Besides, TLR4 is the downstream of IL1R1, and the inactivation of the IL1R1/TLR4 signaling pathway plays a significant role in acquired epilepsy [30]. By inhibiting the IL1R1/TLR4 signaling pathway, the single Ig IL1R-related molecule would block the TLR signaling complex formation as well as tune the action of inflammatory cytokines/chemokines [31]. Further reports came out that overexpression of miR-199b decreased the expressions of IKKβ-NF-κB signaling pathway, TNF-α, and IL-1β in acute spinal cord injury [32].

We also established that the overexpression of miR-495 inhibited cell apoptosis and promoted cell viability through suppression of the TLR4 signaling pathway. Up-regulating miR-483-3p allows a decrease in endothelial progenitor cells (EPCs) migration and tube formation, but increase in apoptosis in LEDVT [1]. Besides, up-regulated miR-495 induces apoptosis while repressing proliferation of gastric cancer cells by targetting the gene of phosphatase of regenerating liver-3 [12]. miR-495 has also been found to directly inhibit E-cadherin expression, hereby promoting cell invasion along with suppressing regulated in development and DNA damage response 1 (REDD1) expression in order to elevate cell proliferation in hypoxia through the process of post-transcriptional mechanism [23]. The aforementioned detailed studies illustrated miR-495 as being a significant factor for both regulation of the proliferation as well as the apoptosis of cancer cells [12,29]. Overexpression of miR-335-5p may suppress the occurrence and development of LEDVT in rats by repressing the activation of the TLR4 signaling pathway by targetted inhibition of PAI-1 [9]. It had also been revealed that the overall effect of the key pro-inflammatory cytokine IL-1 on coagulation and fibrinolysis is pro-thrombotic [33]. IL1R1/IL-1β enhanced both megakaryocyte and platelet functions, thus facilitating a prothrombotic environment and potentially leading to the development of atherothrombotic disease [34]. Therefore, overexpression of miR-495 inhibited the occurrence and development of thrombosis of FV tissues through down-regulation of the IL1R1 expression. It also shows us that IL1R1 not only participates in the formation of DVT, but also has a great correlation with DVT patients who have a tumor, providing a detection index for the diagnosis and prevention of the disease.

## Conclusion

To summarize, the miR-495 expression was down-regulated in LEDVT, while IL1R1 was up-regulated. MiR-495 inhibited both *IL1R1* mRNA and protein expressions by binding to 3′-UTR. Overexpression of miR-495 significantly modulated LEDVT biology by promoting FV endothelial cell proliferation and inhibiting the apoptosis through direct regulation of IL1R1 expression via the TLR4 signaling pathway. Both the effects and mechanisms of miR-495 on LEDVT provide a potential treatment. However, whether the overexpressed miR-495 attenuates LEDVT patients without side effect are needed to further our investigations.

## Supporting information

**supplementary Figure:** 

**supplementary Figure:** 

## Abbreviations

FV: femoral vein
GAPDH: glyceraldehyde-3-phosphate dehydrogenase
HE: Hematoxylin–Eosin
IL1R1: interleukin 1 receptor type 1
IL-1β: interleukin 1 β
LEDVT: lower extremity deep vein thrombosis
NC: negative control
NF-κB p65: nuclear factor-κ B p65
OD: optical density
PI: propidium iodide
Rac1: Rac family small GTPase 1
RT-qPCR: reverse-transcription quantitative PCR
TBST: TBS with Tween 20
TLR: toll-like receptor 4
VEC: vascular endothelial cell
