# Expression of Concern: Effect of microRNA-495 on lower extremity deep vein thrombosis through the TLR4 signaling pathway by regulation of IL1R1

**DOI:** 10.1042/BSR-2018-0598_EOC

**Published:** 2022-05-30

**Keywords:** Cell cycle, Femoral vein, IL1R1, Lower extremity deep vein thrombosis, MicroRNA-495, TLR4 signaling pathway

The Editorial Office has been made aware of potential issues surrounding the scientific validity of this paper, including suspected nonsensical data in Figure 6A, a duplication within Figure 2A between the CD31 and CD34 LEDVT panels, and western blots in Figures 4C and 8E that appear to have no background. The authors have been contacted with regards to these concerns but have currently not responded to the Journal's queries.

