# Supplementary figures and images for: Effect of miR-495 on lower extremity deep vein thrombosis through the TLR4 signaling pathway by regulation of IL1R1
Source: Biosci Rep. 2018 Dec 21;38(6):BSR20180598. doi: 10.1042/BSR20180598 (PMC6435557; doi:10.1042/BSR20180598)

**A**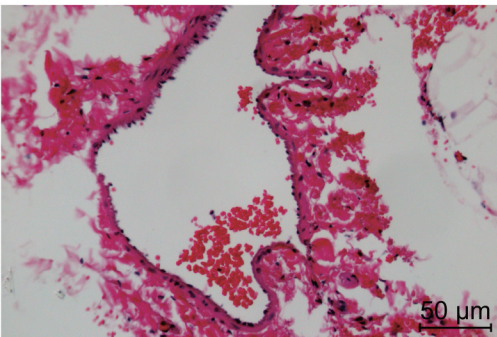

Normal

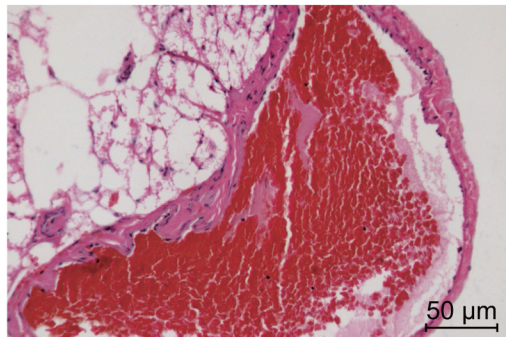

DVT

**B**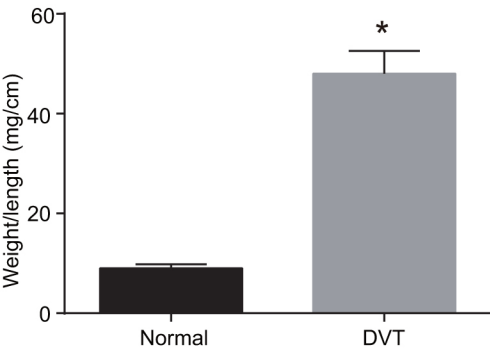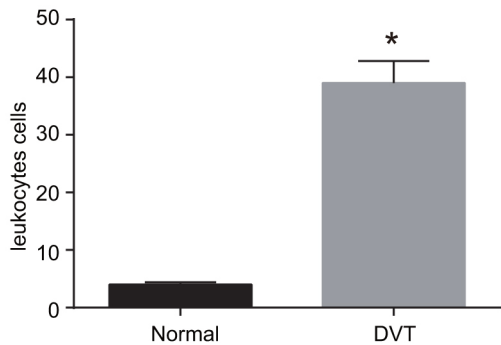

**A**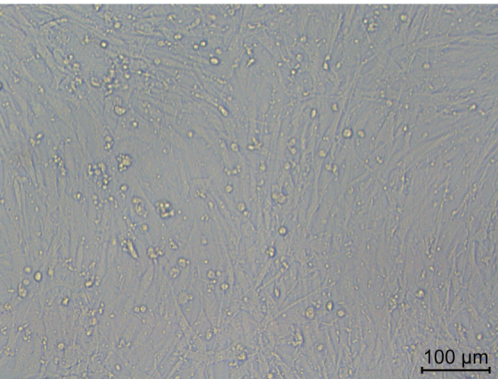**B**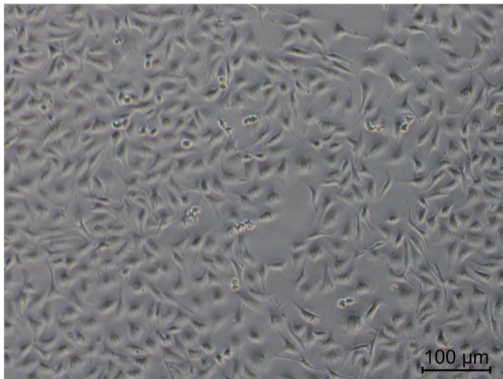

Supplement: Supplementary file 1 [file bsr20180598_Supp1.pdf]
